# Majocchi's granuloma—A multicenter retrospective cohort study

**DOI:** 10.1016/j.jdin.2023.08.010

**Published:** 2023-08-23

**Authors:** Ryan B. Khodadadi, Zachary A. Yetmar, Carmen M. Montagnon, Emma F. Johnson, Omar M. Abu Saleh

**Affiliations:** a Division of Public Health, Infectious Diseases, and Occupational Medicine, Mayo Clinic, Rochester, Minnesota; b Department of Dermatology, Mayo Clinic, Rochester, Minnesota; c Department of Laboratory Medicine and Pathology, Mayo Clinic, Rochester, Minnesota

**Keywords:** antifungal therapy, deep dermatophyte infection, Majocchi granuloma

## Abstract

**Background:**

Majocchi's granuloma (MG) is an uncommon deep fungal folliculitis predominantly caused by dermatophytes. Given the rarity of this condition, available data regarding predisposing comorbidities/risk factors, clinical characteristics, offending microbiologic pathogens, diagnostics, pathologic findings, and treatment approaches has been inferred from historical cases.

**Objectives:**

To review our institutional experience with MG.

**Methods:**

We retrospectively analyzed a multicenter cohort of adult patients diagnosed with MG between 1992 and 2022.

**Results:**

We analyzed 147 patients with MG, 105 of which were male with a median age of 55.6 years. Immunosuppressant and topical corticosteroid use were common prior to development of MG. Dermatologic lesions and their sites of involvement did not differ based on the immune status of patients. *Trichophyton rubrum* was the most common causative pathogen of MG, in addition to other dermatophytes. Treatment duration for all prescribed agents was median 31.5 days with oral terbinafine being the most frequently utilized agent. Clinical resolution was achieved in 96.6% of cases.

**Limitations:**

Retrospective, nonrandomized study.

**Conclusions:**

Although rare and clinically variable in presentation, diagnosis of MG often requires histopathologic confirmation to subsequently direct prolonged treatment with systemic antifungal therapy for mycological cure.

**Capsule Summary**

• Majocchi's granuloma is a rare deep fungal infection with most knowledge of this condition limited to case reports or small case series.
• Majocchi's granuloma presentation is clinically variable, requiring histopathologic examination for diagnostic confirmation to direct prolonged systemic antifungal therapy for management of this condition.

## Background

Majocchi's granuloma (MG) is an uncommon deep fungal folliculitis most commonly caused by dermatophytes.^1^ Dermatophytes consist of 3 genera of fungi (*Microsporum, Trichophyton*, and *Epidermophyton*) which have the ability to invade and multiply within keratinized tissue.^2^^,^^3^ They are also the cause of the more common superficial dermatophytoses including tinea pedis and corporis.^4^

MG often follows trauma/disruption to the skin barrier or occlusion of hair follicles and can affect both immunosuppressed and immunocompetent individuals.^5^^,^^6^ It is frequently mistaken for a dermatitis, and application of topical steroids allows for progression of the lesion. As the dermatophyte invades the deep follicle and disrupts the follicular wall, a robust inflammatory and granulomatous reaction in the perifollicular dermis or subcutaneous tissue develops. Although the clinical presentation of MG is variable, it most commonly presents as perifollicular pustules, papules, and nodules within an erythematous plaque on the extremities or face.^1^^,^^7^^,^^8^

The “gold standard” for diagnosis of MG is histopathologic examination with hematoxylin and eosin staining, often aided with periodic acid-Schiff (PAS), Grocott-Gomori methenamine silver (GMS) staining, and fungal culture.^1^ Given the deep involvement of follicles, topical antifungals are ineffective and systemic treatment is required, most commonly with terbinafine, itraconazole, or griseofulvin.

To date, epidemiologic, clinical, diagnostic, and treatment data of this rare condition have been confined to small numbers of case reports and case series within the literature.^1^^,^^6^ Given these limitations, we sought to review of our institutional experience with MG, highlighting demographic, clinical, diagnostic, and treatment characteristics as well as short-term outcomes.

## Methods

### Study design

We conducted a retrospective cohort study of adult patients diagnosed with MG by searching archived records for terms including “Majocchi,” “Majocchi’s granuloma,” “Majocchi granuloma,” “trichophytic granuloma,” “dermatophytic granuloma,” and “dermatophyte folliculitis” between September 1992 and September 2022 at Mayo Clinic campuses in Arizona, Florida, Minnesota, and Wisconsin.

**Fig 1:**
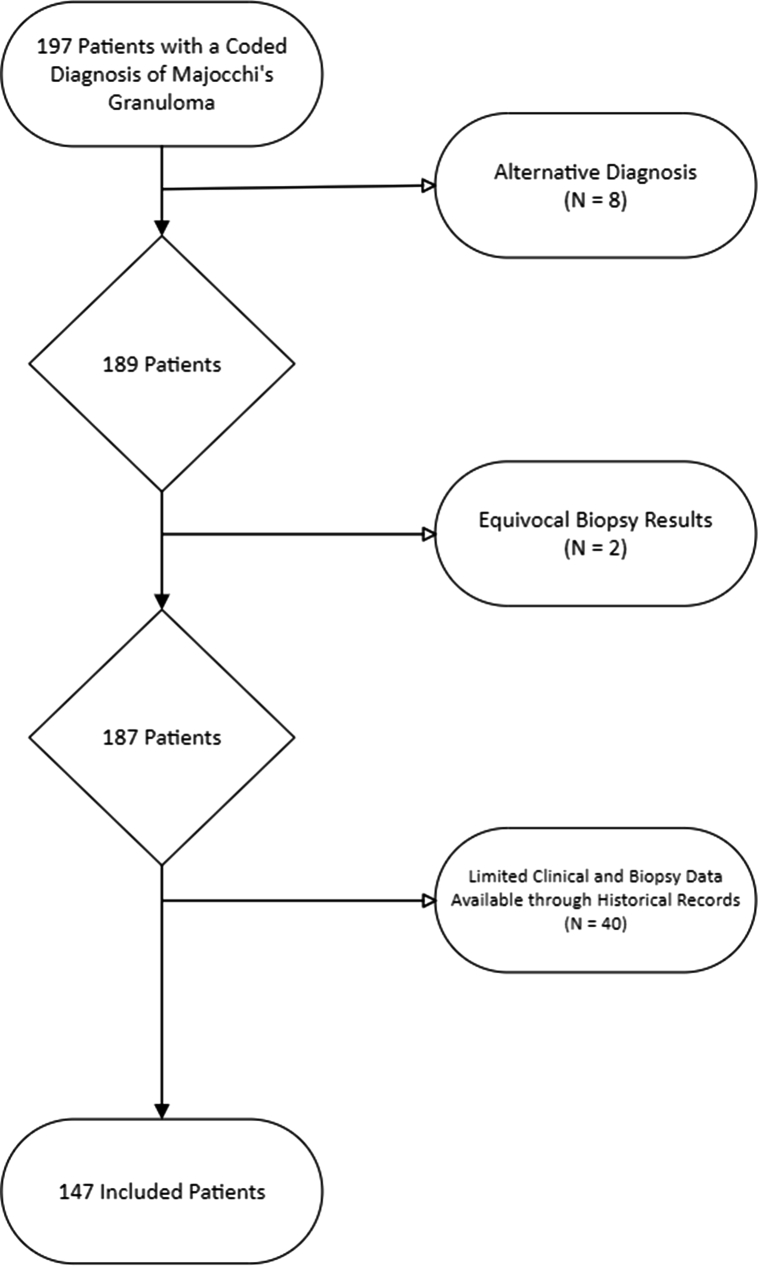
Flow diagram showing exclusion of patients from the final cohort.

Inclusion criteria were age ≥18 years and a confirmed diagnosis of MG. Patients who were found to have an alternative diagnosis, equivocal biopsy results, limited clinical/biopsy information available through historical records, or lacked research authorization per Minnesota statute were excluded (Fig 1). Data from patients who met inclusion criteria were extracted to a prespecified form and were collected and managed utilizing REDCap electronic data capture tools hosted at Mayo Clinic.^9^^,^^10^ Data abstracted included demographics, comorbidities, clinical, diagnostic, microbiologic, and, biopsy characteristics, treatment details, and outcomes. The Charlson comorbidity index was calculated with age adjustment.^11^ The Mayo Clinic Institutional Review Board reviewed the study protocol and granted it exempt status (#22-008957).

### Microbiology

Dermal biopsy tissue was processed and then sent for fungal culture as per standard operating procedure in our microbiology laboratory. Following processing, specimens were plated for the isolation of fungi and examined daily for a standardized duration and incubation temperature.

If plates demonstrated growth, further workup starting with microscopic examination and subculturing were performed onto appropriate media and isolates were submitted for additional testing while further identification and phenotypic testing were in process. Identification was performed by matrix-assisted laser desorption/ionization–time of flight and/or a combination of morphological, biochemical characteristics, and 16S/D2 DNA sequencing.

Following identification, yeast isolates underwent phenotypic antimicrobial susceptibility testing (AST) by microbroth dilution in accordance with Clinical and Laboratory Standards Institute guidelines on Antifungal Susceptibility Testing of Yeasts for species-specific testing and reporting criteria.^12^ Results were interpreted using minimum inhibitory concentration breakpoints and interpretive categories as reported and outlined in Clinical and Laboratory Standards Institute guidelines.

Testing for filamentous fungi was conducted in a similar fashion internally at the Mayo Clinic Microbiology Laboratory (Rochester, MN) until 2020.^13^^,^^14^ Since then, filamentous fungi in addition to dermatophyte AST has been externally referred to the University of Texas at San Antonio Fungal Testing Laboratory (San Antonio, TX).

### Statistical analysis

Continuous variables are presented as mean (standard deviation [SD]) or median (interquartile range [IQR]), as appropriate. Categorical variables are presented as number (percentage). All analyses were performed using BlueSky Statistics version 7.40 software (BlueSky Statistics LLC).

## Results

### Cohort baseline and presenting clinical characteristics

One hundred forty-seven patients with MG met inclusion criteria with all patients being evaluated by a dermatologist (Fig 1). Most patients were male (*n* = 105; 71.4%) with a median age of 55.6 years. Immunosuppressant use prior to presentation use was noted in 27.2% of patients with the most common agent being systemic corticosteroids (*n* = 24, 16.3%) with a prednisone equivalent dose of 20.0 mg (IQR 6.9 mg, 30.0 mg). Topical corticosteroid use prior to dermatologic evaluation was noted in 49% of patients with medium potency being the most frequently prescribed group (*n* = 23, 34.8%). Thirty-nine (27.3%) patients were prescribed either topical or systemic antifungal prior to dermatologic evaluation with a median duration 21.0 days of use. Ketoconazole was the most common topical antifungal agent (*n* = 5, 3.4%) whereas terbinafine was the most prescribed systemic antifungal agent (*n* = 5, 3.4%). Further information regarding the cohort’s baseline characteristics is outlined in Table I.

**Table I:** Baseline characteristics of 147 patients with Majocchi granuloma

Variable	Total (*N* = 147)
Age, y	55.6 (43.1, 70.8)
Male sex	105 (71.4)
Race	
White	138 (93.9)
Black or African American	6 (4.1)
Asian	2 (1.4)
Other	1 (0.7)
Ethnicity	
Not Hispanic or Latino	134 (91.2)
Hispanic or Latino	5 (3.4)
Unknown	8 (5.4)
Charlson comorbidity index	2.0 (0.0, 4.0)
Connective tissue disease	11 (7.5)
Diabetes mellitus	14 (9.5)
Chronic kidney disease∗	13 (8.8)
Active malignancy	17 (11.5)
Severe liver disease	4 (2.7)
HIV/AIDS	1 (0.7)
Solid organ transplant	2 (1.4)
Hematopoietic stem cell transplant	1 (0.7)
Other immunocompromising condition†	2 (1.4)
Immunosuppressant use	40 (27.2)
Systemic corticosteroid	24 (16.3)
Prednisone equivalent dose (mg)	20.0 (6.9, 30.0)
Chemotherapy	5 (3.4)
TNF-α inhibitor	4 (2.7)
Other immunosuppression‡	7 (4.8)
Prior topical corticosteroid use	72 (49)
Super-high potency	18 (27.3)
High potency	22 (33.3)
Medium potency	23 (34.8)
Lower-mid potency	2 (3.0)
Least potent	1 (1.5)
Unknown potency	6 (4.1)
Prior antifungal use	39 (27.3)
Topical	
Ketoconazole	5 (3.4)
Clotrimazole	4 (2.7)
Terbinafine	3 (2.0)
Spectazole	2 (1.4)
Nystatin	2 (1.4)
Econazole	1 (0.7)
Tolnaftate	1 (0.7)
Oral/systemic	
Terbinafine	5 (3.4)
Fluconazole	2 (1.4)
Itraconazole	1 (.7)
Griseofulvin	1 (.7)
Other antifungal	6 (4.1)
Unknown antifungal agent	7 (4.8)
Antifungal duration prior to dermatologic evaluation, days	21.0 (13.0, 36.0)

Data are *N* (%) or median (interquartile range).

∗ Chronic kidney disease was defined as a baseline estimated glomerular filtration rate <60 mL/min/1.73 m^2^, as calculated by the 2021 Chronic Kidney Disease Epidemiology Collaboration equation.

† Other immunosuppressing conditions include autoimmune pancreatitis (1) and bullous pemphigoid on prednisone (1).

‡ Other immunosuppression include antithymocyte globulin (1), intramuscular corticosteroids (1), intravenous immunoglobulin (1), rituximab (2), methotrexate (1), and risankizumab (1).

The average time from symptom onset to diagnosis was 44.0 days (IQR 20.0, 70.0). Twenty-one patients (14.3%) endorsed prior cutaneous trauma at the time of clinical evaluation with the most common mechanism being a scratch/excoriation injury (*n* = 12, 8.2%). Twelve patients reported a coexisting fungal infection at the time of MG diagnosis (8.2%). Four (2.7%) patients had a household contact with a concurrent fungal infection. Zoonotic exposure history was notable in 17 (11.6%) patients, with animal contact with dogs (*n* = 7, 4.8%) and cattle (*n* = 6, 4.1%) being the most common. Perifollicular papules (*n* = 78, 53.1%) were the most frequently observed dermatologic lesions followed by plaques (*n* = 68, 46.3%). Patients were noted to have a median of 1 lesion (1.0, 7.0) with a mean lesion size of 4.0 cm (SD 3.0). The upper extremities were the most affected dermatologic site of involvement (*n* = 64, 43.5%) followed the lower extremities (*n* = 56, 38.1%). Additional information regarding presenting clinical characteristics can be found in Table II.

**Table II:** Clinical, diagnostic, and microbiological characteristics

Variable	Total (*N* = 147)
Time from symptom onset to diagnosis, days (*n* = 143)	44.0 (20.0, 70.0)
Prior cutaneous trauma	21 (14.3)
Scratch/excoriation	12 (8.2)
Penetrating wound	4 (2.7)
Shaving	2 (1.4)
Other	3 (2.0)
Coexisting fungal infection at diagnosis	12 (8.2)
Household contact with a reported fungal infection	4 (2.7)
Animal exposure	17 (11.6)
Dogs	7 (4.8)
Cattle	6 (4.1)
Cats	5 (3.4)
Birds	2 (1.4)
Horses	1 (0.7)
Beavers	1 (0.7)
Guinea pigs	1 (0.7)
Deer	1 (0.7)
Number of lesions	1.0 (1.0, 7.0)
Lesion size (cm), mean (SD)	4.0 (3.0)
Type of lesion	
Perifollicular papules	78 (53.1)
Plaque	68 (46.3)
Pustules	30 (20.4)
Nodules	23 (15.6)
Patch	22 (15.0)
Other	1 (0.7)
Sites of involvement	
Upper extremities	64 (43.5)
Lower extremities	56 (38.1)
Trunk	24 (16.3)
Face	16 (10.9)
Scalp	3 (2.0)
Neck	6 (4.1)
Genital region	7 (4.8)
KOH preparation performed	66 (45.2)
Positive	24 (36.4)
Negative	39 (59.1)
Equivocal	3 (4.5)
Cultures performed	70 (47.9)
*Trichophyton rubrum*	26 (37.1)
*Trichophyton verrucosum*	9 (12.9)
*Trichophyton* species (not further identified)	8 (11.4)
*Trichophyton tonsurans*	4 (5.7)
*Trichophyton mentagrophytes*	3 (4.3)
*Microsporum canis*	2 (2.9)
*Alternaria* species	1 (1.4)
*Candida albicans*	1 (1.4)
*Microsporum gypseum*	1 (1.4)
*Trichophyton violaceum*	1 (1.4)
No growth	14 (20.0)

Data are *N* (%) or median (interquartile range) unless otherwise specified.

*KOH*, Potassium hydroxide.

### Diagnostic, microbiologic, and biopsy characteristics

Sixty-six (45.2%) patients underwent skin scraping of lesions of concern for potassium hydroxide (KOH) preparation with only 24 (36.4%) procedures positive for fungal elements. Of the patients who had a negative KOH, 13 of them were treated with antifungal therapy at the time of initial clinical. KOH preparation was positive in 9 patients with prior antifungal exposure.

Fungal cultures were obtained at the time of skin biopsy in 70 (47.9%) patients. The most common pathogen isolated was *T rubrum* (*n* = 26) followed by *Trichophyton verrucosum* (*n* = 9). Other *Trichophyton* species were identified on fungal culture (*n* = 16) which are outlined in Table II.

Additional dermatophytes and fungi isolated from fungal cultures from skin biopsy tissue were *Microsporum canis* (*n* = 2) and *Microsporum gypseum* (*n* = 1). *Alternaria* species (*n* = 1) and *Candida albicans* (*n* = 1) were also found on fungal culture. Of the fungal cultures that demonstrated no growth (*n* = 14), 4 patients were exposed to antifungal therapy (3 topical and 1 unknown) for a median duration of 18.5 days (IQR 7.0, 30.0) prior to dermatologic evaluation, possibly affecting culture yield in this clinical context. Dedicated antifungal AST was performed by the aforementioned outside referral microbiology laboratory for 1 patient who had a susceptible *T rubrum* isolate to fluconazole.

**Fig 2:**
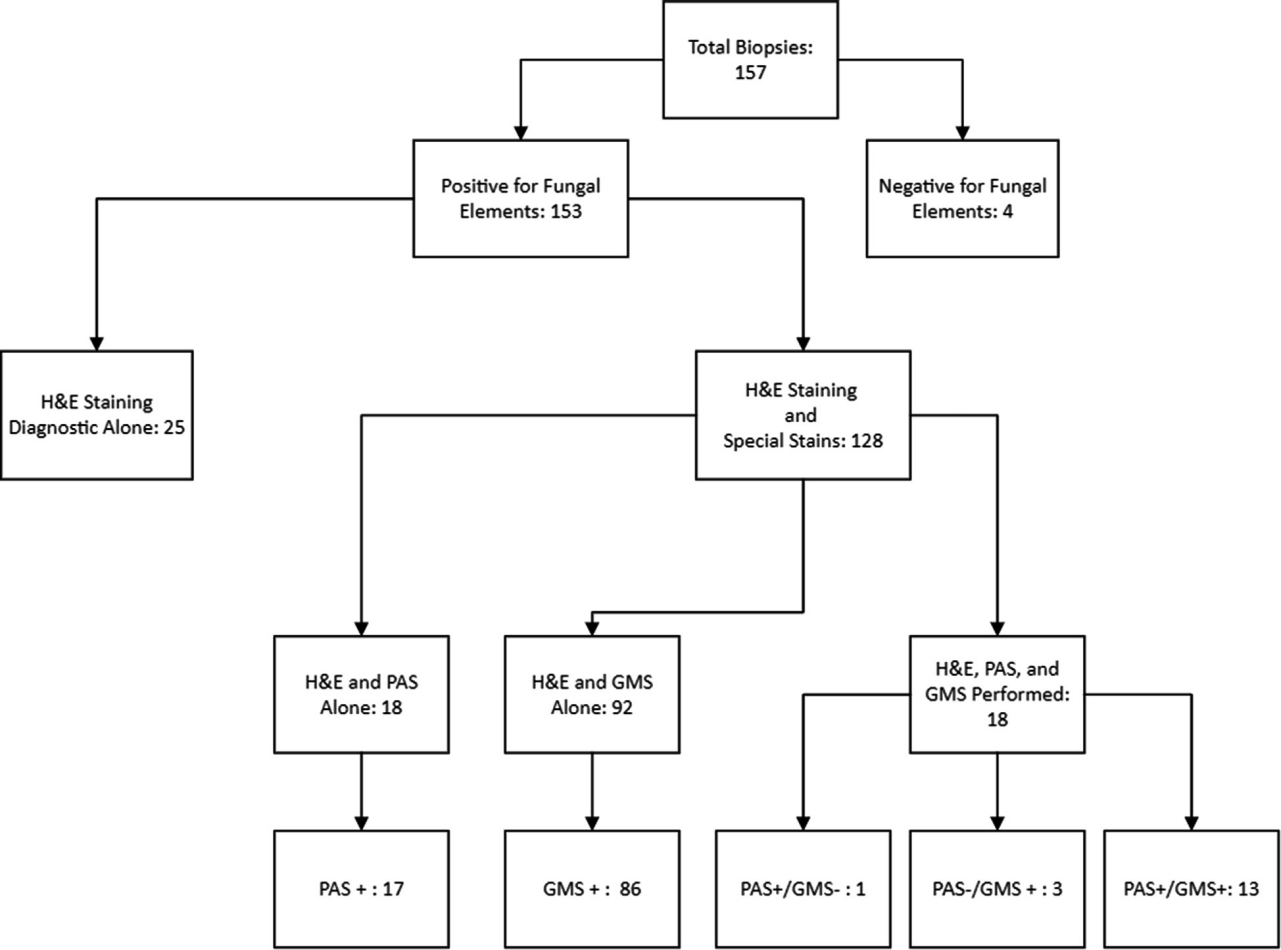
Biopsy characteristics. *GMS*, Grocott-Gomori methenamine silver; *H&E*, hematoxylin-eosin; *PAS*, periodic acid-Schiff.

One hundred fifty-seven skin biopsies were performed on 147 patients with MG. Fungal elements were identified in 153 biopsies (*n* = 158, 96.8%), confirming the diagnosis of MG. Of the 4 biopsies that did not reveal fungal elements on histopathology, 3 were confirmed by the presence of a culprit organism on culture and 1 was confirmed by a positive KOH stain prior to biopsy. Additional information including the rate of fungal identification on hematoxylin and eosin, PAS, and GMS stains available from archived histopathologic reports are reported in Fig 2. Notably, of the 18 biopsies that had both GMS and PAS staining performed, 16 (88.8%) were GMS positive whereas 14 (77.7%) were PAS positive.

### Treatment characteristics and outcomes

Of the total cohort of 147 patients with MG, treatment data were available for 141 patients (96.6%). From this subset, 137 (97.2%) received antifungal therapy. The most common antifungal agent utilized was daily oral terbinafine (*n* = 107, 78.1%) followed by oral itraconazole daily (*n* = 8; 5.8%) and oral fluconazole (*n* = 7; 5.1%). Nineteen patients received oral antifungal therapy in conjunction with topical antifungals whereas 14 patients received topical therapy exclusively. Further information is available in Table III.

**Table III:** Treatment characteristics and outcomes

Variable	Overall (*N* = 141)
Treatment prescribed (*N* = 141)	137 (97.2)
Antifungal agent used (*N* = 137)	
Oral terbinafine	107 (78.1)
Oral itraconazole	8 (5.8)
Oral fluconazole	7 (5.1)
Oral griseofulvin	3 (2.2)
Oral ketoconazole	2 (1.4)
Oral voriconazole	1 (.7)
Topical econazole	8 (5.8)
Topical terbinafine	8 (5.8)
Topical ketoconazole	5 (3.6)
Other topical antifungal agent∗	6 (4.4)
Other†	8 (5.8)
Treatment length (*N* = 132)	31.500 (28.000, 54.000)
Toxicity experienced during treatment (*N* = 118)	6 (5.1)
Resolution of lesions (*N* = 118)	114 (96.6)

Data are *N* (%), mean (standard deviation), or median (interquartile range).

∗ Topical ciclopirox, topical clotrimazole, and topical naftifine hydrochloride.

† Topical metronidazole, oral cephalexin, topical clobetasol, and topical triamcinolone.

Irrespective of regimen or route, median treatment duration was 31.5 days (IQR 28.0, 54.0) with documented clinical resolution in 114 of 118 patients at follow-up. Of the patients who failed to achieve clinical resolution, all were immunocompetent and 3 of 4 patients received antifungal therapy all with oral terbinafine with a mean treatment duration of 98.0 days (IQR 41.0, 136.5) without side effects or toxicity despite prolonged exposure to antifungal therapy.

Transaminitis on routine outpatient antimicrobial monitoring was observed in 6 patients, all of whom received treatment for MG with oral terbinafine, with 1 patient being treated with oral itraconazole in combination as part of their treatment course. The average duration of antifungal therapy within this subset of patients was 48.8 days (SD 21.6 days). Additionally, within this cohort, 2 patients had history of moderate/severe liver disease but had normal liver function testing at baseline.

## Discussion

Our study describes baseline demographics, comorbidities, clinical characteristics, diagnostic/microbiologic findings, treatment characteristics and outcomes of patients with MG who underwent comprehensive dermatologic evaluation at our institution. In 2018, a comprehensive review of published cases of MG from 1883 to 2017 revealed a total of 112 reported cases worldwide.^1^ Since then, <20 additional cases have been reported in addition to 2 centers describing their cases of MG.^6^^,^^15^ To date, our study is the single largest cohort of patients with MG in the literature.

This study demonstrated several notable findings. Among patients who were diagnosed with MG, most were immunocompetent males, consistent with the epidemiology of this disease throughout the literature.^1^ There were 40 patients who either had an immunocompromising condition or were concurrently on immunosuppressing medications, but there were no cases of disseminated disease within this subset of patients or our entire cohort. This includes patients with a solid organ transplant, which has previously been associated as a risk factor for dissemination.^5^^,^^16^ As highlighted in other cases and reviews, topical corticosteroids use prior to diagnosis of MG was common in our study and may be a predisposing factor for the development of MG. Other reports have found TNF-α inhibitors as a predisposing factor which we saw in only 4 patients.^17^^,^^18^

The most common dermatologic lesions identified at initial assessment were perifollicular papules and plaques localized to the upper and lower extremities. There was no observed difference among patients with immunocompromising conditions in terms of lesion appearance and distribution, differing from classically described findings related to MG.^1^^,^^7^

As part of a diagnostic workup, KOH preparation alone, although informative in real-time during clinical assessment if positive, is unreliable for diagnosis of MG given the superficial nature of the procedure. This is especially true for patients with recent exposure to topical/systemic antifungal therapy, underscoring the need for biopsy when clinical suspicion is heightened for MG.^1^ Interestingly, biopsy reports from MG cases in our cohort revealed a higher positivity rate for hematoxylin and eosin and GMS staining as compared with PAS stains, despite PAS staining being the preferred method of staining for histopathologic examination.^19^^,^^20^

Dermatophytes were the predominant pathogens identified on fungal culture as the cause of MG. Similarly, *T rubrum* as reported throughout the literature was also found to be the most common culprit dermatophyte in our cohort.^1^^,^^7^ An association of exposure to cattle with *T verrucosum* and cases of guinea pig exposure linked to MG were also observed in our cohort, congruent with published data on these zoonotic exposures.^21^, ^22^, ^23^ Although rare, alternative organisms have been described as the cause of MG, most commonly secondary to *Aspergillus* species.^8^ This differs from our findings of nondermatophyte cases of MG which included deep dermal cultures positive for *Alternaria* species and *C albican*s.

Among patients who were diagnosed with MG, oral terbinafine was most commonly chosen for definitive therapy with a median treatment duration of 31.5 days resulting in subsequent clinical resolution, rarely associated with toxicity with limited drug-drug interactions. Although there is no consensus regarding treatment duration of MG, the median duration of treatment differs drastically from a recent retrospective study from a single center focused on treatment with mycological cure achieved at 4.38 months for 13 patients treated with oral itraconazole whereas treatment with oral terbinafine resulted in resolution in 3.48 months.^24^ Nevertheless, many patients in our study required longer durations of therapy, and underscored the individualized nature of treatment for MG and importance of follow-up visits to determine adequate response. Overall, courses of 30 days or less appeared to be adequate for many patients.

Strengths of this study include the volume cases of MG evaluated compared with similar descriptive studies in the literature. Additionally, all included patients were evaluated by a dermatologist at the time of presentation prompting biopsy to establish the diagnosis. Subsequent treatment, outpatient antimicrobial therapy monitoring, and follow-up in the absence of formalized treatment guidelines were also consistent given the institutional practice model within the Mayo Clinic Department of Dermatology.

Our study has several limitations worth noting. First, this study was conducted retrospectively and is subject to potential sources of bias and confounding. Second, given the medical complexity of patients seen at our institution, this study may be susceptible to referral bias, possibly limiting the generalizability of results to other patient populations. Further, there is a lack of ethnic diversity this cohort and therefore application of study findings to skin of color may be limited. Third, biopsy characteristics were confined to the data available in histopathologic archived reports and thus additional dermatopathologic characterization of associated findings are limited. Fourth, less than half of biopsies had dedicated fungal culture, impairing our ability to further supplement microbiologic etiologies of MG. Fifth, despite the availability of dermatophyte AST internally at Mayo Clinic until 2020, only one isolate had testing performed prior to aid in antifungal selection. Testing is not routinely performed in microbiology laboratories due to time, cost, and technical difficulties. However, limited susceptibility data exists in the setting of emerging worldwide treatment-resistant superficial dermatophytoses to commonly prescribed agents such as terbinafine, highlighting the need for additional diagnostics to further direct therapy in recalcitrant superficial tinea infections as well as deeper infections including MG such as real-time polymerase chain reaction.^25^, ^26^, ^27^, ^28^

## Conclusion

In summary, this study outlines a detailed review of our institutional experience with biopsy-confirmed cases of MG, highlighting clinical, diagnostic, and treatment characteristics as well as short-term outcomes. KOH staining alone did not appear sufficient to confirm the diagnosis and we would recommend dedicated biopsy for dermatopathologic examination when there is clinical suspicion for MG to confirm the diagnosis. In our experience, these infections generally responded well to courses of therapy of a median 31.5 days, though many patients required prolonged courses of antifungal treatment and therefore it would be reasonable to complete a 4-week course of treatment followed by interval clinical assessment to determine the duration of definitive therapy.

## Conflicts of interest

None disclosed.
